# Equivariant score-based generative diffusion framework for 3D molecules

**DOI:** 10.1186/s12859-024-05810-w

**Published:** 2024-05-30

**Authors:** Hao Zhang, Yang Liu, Xiaoyan Liu, Cheng Wang, Maozu Guo

**Affiliations:** 1 School of Computer Science and Technology, Harbin Institute of Technology, Harbin, 150001 China; 2 School of Electrical and Information Engineering, Beijing University of Civil Engineering and Architecture, Beijing, 100044 China

**Keywords:** 3D molecular generation, Score-based diffusion model, Partial score functions, E(3)-equivariant

## Abstract

**Background:**

Molecular biology is crucial for drug discovery, protein design, and human health. Due to the vastness of the drug-like chemical space, depending on biomedical experts to manually design molecules is exceedingly expensive. Utilizing generative methods with deep learning technology offers an effective approach to streamline the search space for molecular design and save costs. This paper introduces a novel E(3)-equivariant score-based diffusion framework for 3D molecular generation via SDEs, aiming to address the constraints of unified Gaussian diffusion methods. Within the proposed framework EMDS, the complete diffusion is decomposed into separate diffusion processes for distinct components of the molecular feature space, while the modeling processes also capture the complex dependency among these components. Moreover, angle and torsion angle information is integrated into the networks to enhance the modeling of atom coordinates and utilize spatial information more effectively.

**Results:**

Experiments on the widely utilized QM9 dataset demonstrate that our proposed framework significantly outperforms the state-of-the-art methods in all evaluation metrics for 3D molecular generation. Additionally, ablation experiments are conducted to highlight the contribution of key components in our framework, demonstrating the effectiveness of the proposed framework and the performance improvements of incorporating angle and torsion angle information for molecular generation. Finally, the comparative results of distribution show that our method is highly effective in generating molecules that closely resemble the actual scenario.

**Conclusion:**

Through the experiments and comparative results, our framework clearly outperforms previous 3D molecular generation methods, exhibiting significantly better capacity for modeling chemically realistic molecules. The excellent performance of EMDS in 3D molecular generation brings novel and encouraging opportunities for tackling challenging biomedical molecule and protein scenarios.

## Introduction

Molecular science is an interdisciplinary field that encompasses chemistry, pharmacology, materials science, and other related disciplines. Its primary objective is to develop novel concepts and breakthroughs that directly influence human health, energy sustainability, and technological advancement. However, given the immense size of the drug-like chemical space, relying on experts to manually design molecules that are efficient, unique, or possess specific characteristics might be prohibitively costly. In recent years, deep learning methods, including graph representation learning [1, 2], especially deep generative models [3–5], have become increasingly popular in the field of molecular design due to their exceptional ability to learn intricate data distributions and generate novel data samples. By training on a dataset that consists of well-established molecular structures, deep generative models have the ability to generate novel molecules that have structures similar to those in the training dataset, providing an effective method to streamline the search space for molecular design and reduce costs.

Most recently, diffusion models (DMs) [6, 7] have made great progress in image synthesis, exceeding traditional generative models. DMs define a diffusion process that smoothly perturb data towards a known prior distribution, and a neural network is learnt to reverse this perturbation via progressive denoising. Subsequently, the denoising network can generate new data by iteratively removing noise from randomly initialized data. Many researchers have extended and applied the diffusion framework to molecular generation, such as molecular graphs [8, 9], molecular conformations [10–12], and 3D geometric molecules [13–15]. However, the existing molecular generation studies typically employ diffusion methods directly within the atomic feature space, encompassing diverse physical quantities such as atomic types, bond types, and spatial coordinates, along with multiple modes including discrete, integer, and continuous variables. Consequently, the unified Gaussian diffusion model does not offer the optimal solution in this context. Thus, we consider sophisticated decomposition modeling for different variables within the molecular features.

In this paper, we propose a novel E(3)-equivariant score-based diffusion framework for 3D molecular generation, by introducing a diffusion process for 3D geometric structure though stochastic differential equations (SDEs), to overcome the limitations of unified Gaussian diffusion methods. Specifically, the full diffusion is decomposed into separate diffusion processes for distinct components of the molecular feature space, i.e., atom features, bonded adjacency matrix, and spatial coordinates, through the proposed diffusion framework. The proposed framework is referred to as E(3)-equivariant 3D Molecular Diffusion via Stochastic differential equations (EMDS), which describes the perturbation of atom features, adjacency, and coordinates via SDEs. During the modeling process, EMDS also considers the complex dependency of the components in molecular feature space, which is crucial for the generation of real-world molecules, improving the stability and generation performance of the framework. In addition, we consider angle and torsion angle information, which is naturally translation-invariant, to enhance modeling of the molecular coordinate variable and to utilize the spatial information. Subsequent experiments have demonstrated that the incorporation of angle and torsion angle information significantly improves all metrics of the generated molecules, especially molecule stability.

We conduct detailed evaluations of EMDS on QM9 [16], the standard molecule dataset. Experimental results show that EMDS consistently achieves superior performance on all metrics, demonstrating the effectiveness and excellent performance of our model for unconditional 3D molecular generation. Furthermore, we conduct ablation studies and present distribution comparisons between molecules in the test set and molecules generated by EMDS to demonstrate that the proposed method is highly effective for the molecular generation task. We make the dataset and source code publicly available at https://github.com/nclabhzhang/EMDS.

The contributions of our work can be summarized as follows:

1. We propose a novel E(3)-equivariant score-based diffusion framework EMDS for 3D molecular generation, in which the full diffusion is decomposed into separate diffusion processes for distinct components of the molecular feature space, and the complex dependency of these components can be efficiently captured during the modeling process, overcoming the limitations of unified Gaussian diffusion methods.
2. We incorporate the angle and torsion angle information into the diffusion framework by introducing the inter-atomic cosine information into score-based networks, effectively modeling the coordinate variable, exploiting the spatial information, and significantly improving the stability of the generated molecules.
3. We demonstrate the excellent performance of the proposed method EMDS in comparison with several state-of-the-art methods for 3D molecular generation.

## Related work

Deep generative models have recently demonstrated their effectiveness in modeling the density of real-world molecule data for both molecule design and generation [17–20]. As the predominant method for molecular representation, 1D SMILES [21] has been widely utilized in many studies involving sequence-based generative models [22–24]. Due to great advances in graph neural networks, extensive research has been devoted to generating molecules in a 2D format [23, 25–27]. In addition, some research [1, 2] has been conducted on graph representation learning, which has demonstrated encouraging outcomes in fields like drug localization and drug-target interactions. While these studies have the capacity to generate valid molecules and predict drug-related interactions, they neglect the 3D geometric structure information of molecules, which is crucial for molecular design. Some recent methods attempt to generate 3D molecules directly. Gebauer et al. [28, 29] and Luo & Ji [30] generate molecules by sampling atoms from an order-dependent autoregressive distribution, step by step. Satorras et al. [31] propose equivariant normalizing flows to jointly generate molecule features and positions in 3D.

Most recently, DMs [6, 7, 32] have gained great interest as powerful generative models for molecular discovery applications [8, 9, 12]. Furthermore, some research has explored the incorporation of equivariant graph methods into 3D geometric structure generative diffusion models [13, 14, 33]. Some researchers have also begun to explore the use of diffusion methods for tasks such as target generation, conditional generation [34, 35], and linker design [36], among others. While these methods achieve promising results for molecular generation, the multi-modal nature of the variables in the atomic feature space makes the unified Gaussian diffusion framework suboptimal. In our proposed framework, full diffusion is decomposed into diffusion processes of respective components of the molecular feature space to overcome the limitations of unified Gaussian diffusion methods. In addition, in contrast to other diffusion methods, angle and torsion angle information can be captured and learned in our proposed method, improving modeling of molecular coordinates and utilizing spatial information.

## Preliminaries

### Diffusion models

Diffusion models [6, 37] are latent variable models that learn distributions by modeling the reverse of a diffusion process, known as the denoising process. In order to construct diffusion models, it is necessary to first define a forward diffusion process. Given a data point *x*, the forward diffusion process that perturbs data with a sequence of noise $z_{t}$ for $t=0,1,2,...T$ until the marginal distribution matches a known prior distribution, which can be defined by the multivariate normal distribution:

1 $$\begin{aligned} q(z_{t}|x)=\mathcal {N}(z_{t}|\alpha _{t}x_{t},\sigma ^{2}_{t}\mathbf{I}) \end{aligned}$$

where $\alpha _{t}\in \mathbb {R}^{+}$ denotes the degree of signal retention and $\sigma _{t}\in \mathbb {R}^{+}$ denotes the degree of noise addition. Both $\alpha _{t}$ and $\sigma _{t}$ are time-dependent differentiable functions. In general, $\alpha _{t}$ is modelled by a function that smoothly transitions from $\alpha _{0}\approx 0$ towards $\alpha _{T}\approx 1$. A special case of noising process is the variance preserving process, for which $\alpha _{t}=\sqrt{1-\sigma _{t}^{2}}$. By implementing this method, $\alpha _{t}$ and $\sigma _{t}$ guarantee that the prior distribution $q(z_{t})$ adheres to a normal distribution while maintaining a strictly decreasing signal-to-noise ratio of $\alpha _{t}=\sqrt{1-\sigma _{t}^{2}}$. This diffusion process is Markovian, and the entire noising process can be written as:

2 $$\begin{aligned} q(z_{0},z_{1},...,z_{T}|x)=q(z_{0}|x)\prod _{t=1}^{T}q(z_{t}|z_{t-1}) \end{aligned}$$

In contrast to other generative models, the generation process of diffusion models is defined by learning a parameterized reverse denoising process. The denoising neural network $\phi$ learns to predict the clean data *x* in the target data distribution from $z_{t}$. In fact, the variable *x* is unknown during the generative process, with its approximation replaced by the neural network $\hat{x}=\phi (z_{t},t)$. Then the generative transition distribution can be expressed using the approximation $\hat{x}$:

3 $$\begin{aligned} p(z_{t-1}|z_{t})=\mathcal {N}(z_{t-1};\mu _{t}(\hat{x},z_{t}),\rho _{t}^{2}\mathbf{I})) \end{aligned}$$

where $t=T,...,1$, and the initial distribution $p(z_{T})$ is defined as $\mathcal {N}(0,\mathbf{I})$.

As a latent variable model, in diffusion, the forward process $q(z_{0:T}|x)$ can be considered a fixed posterior, to which the reverse process $p(x|z_{0:T})$ is trained to maximize the variational lower bound of the likelihood of *x*:

4 $$\begin{aligned} \text{log}p(x)\ge \mathcal {L}_{0}+\mathcal {L}_{base}+\sum _{t=1}^{T}\mathcal {L}_{t} \end{aligned}$$

where $\mathcal {L}_{0}=\text{log}p(x|z_{0})$ denotes a reconstruction term that matches the likelihood of the data given $z_{0}$, $\mathcal {L}_{base}=-\text{KL}(q(z_{T}|x)\parallel p(z_{T}))$ matches the distance between a standard normal distribution and the final latent variable $q(z_{T}|x)$, and $\mathcal {L}_{t}=-\text{KL}(q(z_{t-1}|x,z_{t})\parallel p(z_{t-1}|z_{t}))$, $t=1,...,T$, denotes denoising matching term.

### Score-based method

However, each KL divergence term $\mathcal {L}_{t}$ in Eq. 4 is difficult to minimize and directly optimizing this objective will suffer from training instability [38]. Specifically, if the reparameterization method and Tweedie’s formula are utilizd, i.e., $z=\alpha _{t}x+\sigma _{t}\varepsilon$ with $\varepsilon \in \mathcal {N}(0,\mathbf{I})$ and $\mathbb {E}[\mu _{t}|z_{t}]= z_{t}+\rho _{t}^{2}\triangledown _{z_{t}}\text{log}p(z_{t})$ for a Gaussian variable $z_{t}\in \mathcal {N}(z_{t};\mu _{t},\rho _{t}^{2})$, then $\hat{x}$ can be expressed as follows:

5 $$\begin{aligned} \hat{x}=\frac{1}{\alpha _{t}}z_{t}-\frac{\sigma _{t}}{\alpha _{t}}\triangledown _{z_{t}}\text{log}p(z_{t}) \end{aligned}$$

Subsequently, the corresponding optimization becomes as follows:

6 $$\begin{aligned} {\underset{\theta }{\text{argmin}}}\mathcal {L}_{t}= {\underset{\theta }{\text{argmin}}}\frac{1}{2\rho _{t}^{2}}\frac{1-\alpha _{t}^{2}}{\alpha _{t}}\left[ \left\| \phi (z_{t},t)-\triangledown _{z_{t}}\text{log}p(z_{t}) \right\| _{2}^{2} \right] \end{aligned}$$

Here, $\phi (z_{t},t)$ is a neural network that learns to predict the score function $\triangledown _{z_{t}}\text{log}p(z_{t})$, which is the gradient of $z_{t}$ in data space, for any arbitrary noise time step *t*, and $\theta$ represents the parameters of the neural network $\phi (z_{t},t)$. In practice, $\mathcal {L}_{base}$ is close to zero when the noising schedule is defined in such a way that $\alpha _{T}\approx 0$. Furthermore, if $\alpha _{0}\approx 1$ and *x* is discrete, then the reconstruction term $\mathcal {L}_{0}$ is close to zero as well. At this point, we observe an explicit connection between the diffusion model and the score-based generative model.

In the following discussion, we will explain how the score-based diffusion model can be extended to encompass an infinite number of time steps. From the perspective of the Markovian Hierarchical Variational Autoencoder (HVAE) [39, 40], this extension can be interpreted as expanding the number of levels to infinity *T*. A stochastic process can describe how data perturbs in continuous time across an infinite number of noise scales, which can be expressed by a SDE. Sampling is accomplished by inverting the SDE, which requires estimating a scoring function for each continuous-valued noise level [7]. Different parameterizations of the SDE describe various perturbation scenarios over time, enabling flexible modeling of the noise process [32].

### Equivariance

Generating 3D molecules presents a significant challenge as the likelihood function of molecular geometry should remain invariant to rotations, translations, and permutations. In order to address this issue, it is necessary to clarify the concepts of invariance and equivariance. A function f is considered equivariant with respect to the action of a group $G$ if, for all $g \in G$, it satisfies the equation $T_{g} \circ f ( x )= f \circ S_{g} ( x ))$, where $S_{g}$ and $T_{g}$ denote transformations for the group element $g$. Following previous work[13], we consider the Euclidean group $E (3)$, comprising translations, rotations, and reflections, for which $S_{g}$ and $T_{g}$ can be represented by a translation ${{\varvec{t}}}$ and an orthogonal matrix $\mathbf{R}$ for the rotated or reflected coordinates.

In molecules, the features $h =( h _{1},..., h _{N})\in \mathbb {R}^{N\times nf}$ are invariant to group transformations, and the coordinates $x =( x _{1},..., x _{N})\in \mathbb {R}^{N\times 3}$ will be affected by rotations, reflections, and translations as $\text{R} x +t=(\text{R} x _{1}+t,...,\text{R} x _{N}+t)$,^1^ where *R* denotes an orthogonal matrix. It is proved that an invariant prior distribution $p(Z_{T})$ and an equivariant neural network to parameterize the transition kernels $p(z_{t-1}|z_{t})$ in the diffusion model ensure the marginal distribution *p*(*x*) is invariant, which is desired for 3D molecular generation [12, 41]. In other words, this requires our learned likelihood to be invariant to roto-translations.

## Methodology

**Fig. 1:**
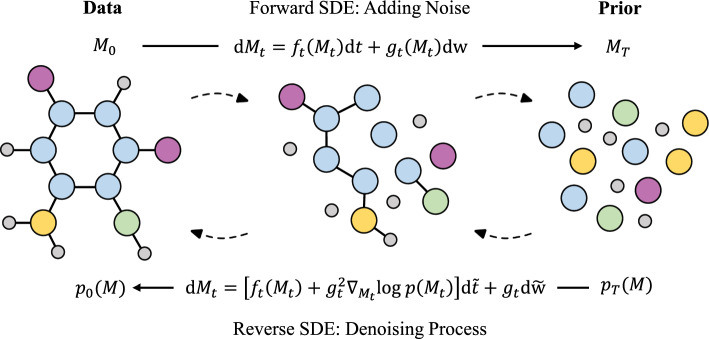
Overview of the proposed framework E(3)-equivariant 3D Molecular Diffusion via SDEs. The model would train 3D molecules, which include atom features, bond types, and spatial coordinates, with a stochastic time step. For the reverse process, the final molecules are generated by denoising the initial state $\mathcal {M} _{T}$ from standard normal noise $\mathcal {N}(0,I)$ gradually with the reverse SDE. Symmetrically, the diffusion process is achieved by adding the noise with the forward SDE until the molecule is degenerated into standard Gaussian noise when the time step is large enough

In this section, we introduce our novel continuous-time E(3)-equivariant score-based diffusion framework for 3D molecular generation through SDEs. An overview of our framework is given in Fig. 1.

### Representation of 3D molecules

A molecule is a collection of atoms connected by chemical bonds, categorized based on the characteristics of the bonds. And the configuration of a molecule can be visualized and depicted in both 2D and 3D formats. The 2D representations focus on depicting the connections between atoms, while the 3D representations showcase the spatial arrangement of these atoms. For a complete description of a molecule *M* with *N* atoms, in this paper, we represent it as a tuple ${{\varvec{M}}}=( {{\varvec{X}}},{{\varvec{P}}},{{\varvec{A}}})$, where ${{\varvec{X}}} \in \mathbb {R}^{N\times k}$ denotes the atom type matrix, *N* is the number of atoms, and *k* is the feature dimension; ${{\varvec{P}}} \in \mathbb {R}^{N\times 3}$ represents the 3D geometric structure, and each row indicates the coordinates of the atom in the Euclidean space; and ${{\varvec{A}}} \in \mathbb {R}^{N\times N}$ denotes the weighted adjacency matrix of the 2D graph representation for chemical bonds. For simplicity, this paper assumes that molecules are fully connected and treats no-bonds as a special edge type. In order to consider the symmetry of the molecule, symmetric edge representations are adopted, i.e., ${{\varvec{A}}}={{\varvec{A}}}^{T}$.

### 3D geometric structure diffusion process

The objective of 3D molecular generation is to synthesize all components, including atoms, bonds, and spatial coordinates, while closely following the distribution of the observed set of molecules. Directly representing the distribution of a three-dimensional molecule is intricate. Simultaneously, employing diffusion methods directly in the atomic feature space is suboptimal, as previously mentioned. Therefore, we consider sophisticated decomposition modeling for different variables within the molecular features and introduce a framework for generating 3D geometric structure based on continuous-time fractions, thereby addressing the problems. Specifically, we propose a novel method of 3D geometric structure diffusion via SDEs, enabling the transformation of molecules into normal noise and vice versa. The method models the decomposition of various variables within molecular features and also captures the complex interdependency among them, which is crucial for the generation of real-world molecules. We first explain the proposed 3D geometric structure diffusion process.

To model the interdependency among ${{\varvec{X}}}$, ${{\varvec{P}}}$, and ${{\varvec{A}}}$, the proposed forward diffusion process of geometric structure should be capable of transforming atom-type features, adjacency matrices, and spatial coordinates into a simple noise distribution. Formally, the diffusion process is represented as the trajectory of random variables ${{\varvec{M}}}_{t}=( {{\varvec{X}}}_{t},{{\varvec{P}}}_{t},{{\varvec{A}}}_{t})$, indexed by a time variable $t\in \left[ 0,T \right]$, where ${{\varvec{M}}}_{0}$ denotes 3D molecules from the data distribution $p_{data}$. Then, the forward diffusion process of 3D geometric structure is modeled by the following $\mathrm {It\hat{o}}$ SDE:

7 $$\begin{aligned} \text{d}{{\varvec{M}}}_{t}=f_{t}({{\varvec{M}}}_{t})\text{d}t+\mathbf{g}_{t}({{\varvec{M}}}_{t})\text{d}\mathrm {\mathbf{w}},\quad t\in \left[ 0,T \right] \end{aligned}$$

where $f_{t}(\cdot ):\mathcal {M}\rightarrow \mathcal {M}$ denotes the linear drift coefficient, $\mathbf{g}_{t}(\cdot ):\mathcal {M}\rightarrow \mathcal {M}\times \mathcal {M}$ denotes the diffusion coefficient, and $\mathrm {\mathbf{w}}$ is the standard Wiener process. Note that, in this paper, function $\mathcal {F}$ subscripted with *t* denotes functions dependent on time: $\mathcal {F}_{t}(\cdot )=\mathcal {F}(\cdot ,t)$. In an intuitive sense, by adding infinitesimal noise $\text{d}\mathrm {\mathbf{w}}$ at each infinitesimal time step $\text{d}t$, the forward diffusion process achieves a smooth transformation of data distribution, i.e., ${{\varvec{X}}}_{0}$, ${{\varvec{P}}}_{0}$, and ${{\varvec{A}}}_{0}$. For the choice of the coefficients $f_{t}$ and $\mathbf{g}_{t}$, it is necessary to make the diffusion sample ${{\varvec{M}}}_{t}$ approximately follow a known prior distribution at the final time horizon *T*, which has a form that can be manipulated to generate samples efficiently, such as the standard normal distribution. For ease of subsequent formulation, $\mathbf{g}_{t}({{\varvec{M}}}_{t})$ will be denoted as the scalar function $g_{t}$.

### Generative denoising process

The denoising process takes the noisy geometric structure as input and aims to predict the corresponding clean structure data. To generate molecules that follow the data distribution, we will reverse the forward diffusion process described in Eq. 7 over time, using the samples from the prior distribution as our initial starting points. It should be noted that the diffusion process in reverse time is also a diffusion process, as the following reverse-time SDE describes:

8 $$\begin{aligned} \text{d}{{\varvec{M}}}_{t}=[f_{t}({{\varvec{M}}}_{t})-g_{t}^{2}\triangledown _{{{\varvec{M}}}_{t}}\text{log}p_{t}({{\varvec{M}}}_{t})]\text{d}\tilde{t}+g_{t}\text{d}\tilde{\mathbf{w}} \end{aligned}$$

where $f_{t}$ and $g_{t}$ are diffusion coefficients, $\text{log}p_{t}({{\varvec{M}}}_{t})$ denotes the marginal distribution of data at time *t*, $\text{d}\tilde{t}$ denotes an infinitesimal negative time step, and $\tilde{\mathbf{w}}$ denotes a reverse-time standard Wiener process.

Directly solving the high-dimensional score term $\triangledown _{{{\varvec{M}}}_{t}}\text{log}p_{t}({{\varvec{M}}}_{t})$ is computationally prohibitive due to the variable ${{\varvec{M}}}_{t}=({{\varvec{X}}}_{t},{{\varvec{P}}}_{t},{{\varvec{A}}}_{t}) \in \mathbb {R}^{N\times K} \times \mathbb {R}^{N\times 3} \times \mathbb {R}^{N\times N}$. Therefore, we propose a reverse-time diffusion approach equivalent to Eq. 8 to circumvent the expensive high-dimensional calculations, which is represented by the following SDEs:

9 $$\begin{aligned}&\text{d}{{\varvec{X}}}_{t}=[f_{1,t}({{\varvec{X}}}_{t})-g_{1,t}^{2}\triangledown _{{{\varvec{X}}}_{t}}\text{log}p_{t}({{\varvec{X}}}_{t},{{\varvec{P}}}_{t},{{\varvec{A}}}_{t})]\text{d}\tilde{t}+g_{1,t}\text{d}\tilde{\mathbf{w}}_{{{\varvec{X}}}} \nonumber \\&\text{d}{{\varvec{P}}}_{t}=[f_{2,t}({{\varvec{P}}}_{t})-g_{2,t}^{2}\triangledown _{{{\varvec{P}}}_{t}}\text{log}p_{t}({{\varvec{X}}}_{t},{{\varvec{P}}}_{t},{{\varvec{A}}}_{t})]\text{d}\tilde{t}+g_{2,t}\text{d}\tilde{\mathbf{w}}_{{{\varvec{P}}}} \nonumber \\&\text{d}{{\varvec{A}}}_{t}=[f_{3,t}({{\varvec{A}}}_{t})-g_{3,t}^{2}\triangledown _{{{\varvec{A}}}_{t}}\text{log}p_{t}({{\varvec{X}}}_{t},{{\varvec{P}}}_{t},{{\varvec{A}}}_{t})]\text{d}\tilde{t}+g_{3,t}\text{d}\tilde{\mathbf{w}}_{{{\varvec{A}}}} \end{aligned}$$

where $f_{1,t}$, $f_{2,t}$, and $f_{3,t}$ denote linear drift coefficients in a reverse-time diffusion process that satisfies $f_{t}({{\varvec{X}}},{{\varvec{P}}},{{\varvec{A}}})=(f_{1,t}({{\varvec{X}}}),f_{2,t}({{\varvec{P}}}),f_{3,t}({{\varvec{A}}}))$, $g_{1,t}$, $g_{2,t}$, and $g_{3,t}$ denote scalar diffusion coefficients, and $\tilde{\mathbf{w}}_{{{\varvec{X}}}}$, $\tilde{\mathbf{w}}_{{{\varvec{P}}}}$, and $\tilde{\mathbf{w}}_{{{\varvec{A}}}}$ are independent reverse-time standard Wiener processes. The inverse-time diffusion process for each component (${{\varvec{X}}}$,${{\varvec{P}}}$ and ${{\varvec{A}}}$) is described separately by the corresponding SDE in Eq. 9, providing a new perspective that characterizes the diffusion of 3D geometric structure as the diffusion of each component over time.

In the SDEs written in Eq. 9, the complete diffusion is decomposed into separate diffusion processes for each component of the molecular feature space to model variables with different characteristics, respectively. Additionally, another property of EMDS is that the diffusion processes are interdependent and related to the gradient of the logarithmic marginal distribution $\triangledown _{{{\varvec{M}}}_{t}}\text{log}p_{t}({{\varvec{M}}}_{t})$, which consists of $\triangledown _{{{\varvec{X}}}_{t}}\text{log}p_{t}({{\varvec{X}}}_{t},{{\varvec{P}}}_{t},{{\varvec{A}}}_{t})$, $\triangledown _{{{\varvec{P}}}_{t}}\text{log}p_{t}({{\varvec{X}}}_{t},{{\varvec{P}}}_{t},{{\varvec{A}}}_{t})$, and $\triangledown _{{{\varvec{A}}}_{t}}\text{log}p_{t}({{\varvec{X}}}_{t},{{\varvec{P}}}_{t},{{\varvec{A}}}_{t})$, also known as the partial score functions [7, 8]. By utilizing partial scores to model the dependency among the components over time, the proposed method can represent the diffusion process of an entire 3D molecule.

If the partial scores for all times *t* in Eq. 9 are available, the SDEs in reverse-time can serve as a generative model for 3D geometric structure. We use the trained score-based networks $s_{\theta ,t}$, $s_{\varphi ,t}$, and $s_{\psi ,t}$ to approximate the inverse-time SDEs as follows:

10 $$\begin{aligned}&\text{d}{{\varvec{X}}}_{t}=[f_{1,t}({{\varvec{X}}}_{t})-g_{1,t}^{2}s_{\theta ,t}({{\varvec{X}}}_{t},{{\varvec{P}}}_{t},{{\varvec{A}}}_{t})]\text{d}\tilde{t}+g_{1,t}\text{d}\tilde{\mathbf{w}}_{{{\varvec{X}}}} \nonumber \\&\text{d}{{\varvec{P}}}_{t}=[f_{2,t}({{\varvec{P}}}_{t})-g_{2,t}^{2}s_{\varphi ,t}({{\varvec{X}}}_{t},{{\varvec{P}}}_{t},{{\varvec{A}}}_{t})]\text{d}\tilde{t}+g_{2,t}\text{d}\tilde{\mathbf{w}}_{{{\varvec{P}}}} \nonumber \\&\text{d}{{\varvec{A}}}_{t}=[f_{3,t}({{\varvec{A}}}_{t})-g_{3,t}^{2}s_{\psi ,t}({{\varvec{X}}}_{t},{{\varvec{P}}}_{t},{{\varvec{A}}}_{t})]\text{d}\tilde{t}+g_{3,t}\text{d}\tilde{\mathbf{w}}_{{{\varvec{A}}}} \end{aligned}$$

where $s_{\theta ,t}$, $s_{\varphi ,t}$, and $s_{\psi ,t}$ are time-dependent score-based networks used to estimate partial score functions, which satisfy $s_{\theta ,t}({{\varvec{M}}}_{t})\approx \triangledown _{{{\varvec{X}}}_{t}}\text{log}p_{t}({{\varvec{M}}}_{t})$, $s_{\varphi ,t}({{\varvec{M}}}_{t})\approx \triangledown _{{{\varvec{P}}}_{t}}\text{log}p_{t}({{\varvec{M}}}_{t})$, and $s_{\psi ,t}({{\varvec{M}}}_{t})\approx \triangledown _{{{\varvec{A}}}_{t}}\text{log}p_{t}({{\varvec{M}}}_{t})$.

After constructing the reverse-time SDE, we can use numerical methods to simulate it to generate samples. Numerical solvers provide approximate trajectories from SDEs. Various numerical methods are available for solving SDEs, including Euler-Maruyama and stochastic Runge–Kutta [42], and all are suitable for generating samples for the reverse-time SDEs. Predictor-Corrector (PC) sampler [7] is adopted to solve the reverse-time SDEs in this paper due to its simplicity and effectiveness. The sampler is able to use a score-based MCMC method like Langevin MCMC [43] to sample from $p_{t}$ and correct the solution provided by the numerical SDE solver. To make our mainline description both complete and concise, we only briefly describe the application of the PC sampler here, as it is not one of our main innovations. Additional details of the PC sampler algorithm for SDEs can be found in [7].

### Optimization objective

The estimation of partial score functions can be achieved through the training of time-dependent score-based networks. The networks need then be trained to minimize the distance to the corresponding ground-truth partial scores. For the minimization of Euclidean distance, we generalize the partial score estimation for a given graph dataset [8] to the 3D geometric structure, and the new objectives can be written as follows:

11 $$\begin{aligned}&\min _{\theta }\mathbb {E}_{t}\left\{ \gamma _{1}(t)\mathbb {E}_{{{\varvec{M}}}_{0}}\mathbb {E}_{{{\varvec{M}}}_{t}|{{\varvec{M}}}_{0}}\left\| s_{\theta ,t}({{\varvec{M}}}_{t})-\triangledown _{{{\varvec{X}}}_{t}}\text{log}p_{t}({{\varvec{M}}}_{t}) \right\| _{2}^{2} \right\} \nonumber \\&\min _{\phi }\mathbb {E}_{t}\left\{ \gamma _{2}(t)\mathbb {E}_{{{\varvec{M}}}_{0}}\mathbb {E}_{{{\varvec{M}}}_{t}|{{\varvec{M}}}_{0}}\left\| s_{\phi ,t}({{\varvec{M}}}_{t})-\triangledown _{{{\varvec{P}}}_{t}}\text{log}p_{t}({{\varvec{M}}}_{t}) \right\| _{2}^{2} \right\} \nonumber \\&\min _{\psi }\mathbb {E}_{t}\left\{ \gamma _{3}(t)\mathbb {E}_{{{\varvec{M}}}_{0}}\mathbb {E}_{{{\varvec{M}}}_{t}|{{\varvec{M}}}_{0}}\left\| s_{\psi ,t}({{\varvec{M}}}_{t})-\triangledown _{{{\varvec{A}}}_{t}}\text{log}p_{t}({{\varvec{M}}}_{t}) \right\| _{2}^{2} \right\} \end{aligned}$$

where $\mathbb {E}_{{{\varvec{M}}}_{0}}$ is taken over the sample ${{\varvec{M}}}_{0}\sim p_{data}$, $\mathbb {E}_{{{\varvec{M}}}_{t}|{{\varvec{M}}}_{0}}$ is taken over the sample ${{\varvec{M}}}_{t}\sim p_{0t}({{\varvec{M}}}_{t}|{{\varvec{M}}}_{0})$, $p_{0t}({{\varvec{M}}}_{t}|{{\varvec{M}}}_{0})$ represents the transition distribution from $p_{0}$ to $p_{t}$, $\gamma _{1}(t)$, $\gamma _{2}(t)$, and $\gamma _{3}(t)$ denote the loss weighted functions, and *t* is uniformly sampled from range [0, *T*].

However, the optimization objective described in Eq. 11 cannot be directly applied in the training process since we usually do not have access to the ground-truth partial scores in practice. The current method [8] demonstrates that it is possible to replace the partial scores through denoising score matching [7], and then the new equivalent objective can be written as follows:

12 $$\begin{aligned}&\min _{\theta }\mathbb {E}_{t}\left\{ \gamma _{1}(t)\mathbb {E}_{{{\varvec{M}}}_{0}}\mathbb {E}_{{{\varvec{M}}}_{t}|{{\varvec{M}}}_{0}}\left\| s_{\theta ,t}({{\varvec{M}}}_{t})-\triangledown _{{{\varvec{X}}}_{t}}\text{log}p_{t}({{\varvec{M}}}_{t}|{{\varvec{M}}}_{0}) \right\| _{2}^{2} \right\} \nonumber \\&\min _{\phi }\mathbb {E}_{t}\left\{ \gamma _{2}(t)\mathbb {E}_{{{\varvec{M}}}_{0}}\mathbb {E}_{{{\varvec{M}}}_{t}|{{\varvec{M}}}_{0}}\left\| s_{\phi ,t}({{\varvec{M}}}_{t})-\triangledown _{{{\varvec{P}}}_{t}}\text{log}p_{t}({{\varvec{M}}}_{t}|{{\varvec{M}}}_{0}) \right\| _{2}^{2} \right\} \nonumber \\&\min _{\psi }\mathbb {E}_{t}\left\{ \gamma _{3}(t)\mathbb {E}_{{{\varvec{M}}}_{0}}\mathbb {E}_{{{\varvec{M}}}_{t}|{{\varvec{M}}}_{0}}\left\| s_{\psi ,t}({{\varvec{M}}}_{t})-\triangledown _{{{\varvec{A}}}_{t}}\text{log}p_{t}({{\varvec{M}}}_{t}|{{\varvec{M}}}_{0}) \right\| _{2}^{2} \right\} \end{aligned}$$

Due to the fact that the drift coefficient $f_{t}(\cdot )$ in Eq. 7 is linear, the transition distribution $p_{0t}({{\varvec{M}}}_{t}|{{\varvec{M}}}_{0})$ can be separated as $p_{0t}({{\varvec{M}}}_{t}|{{\varvec{M}}}_{0}) =p_{0t}({{\varvec{X}}}_{t}|{{\varvec{X}}}_{0})p_{0t}({{\varvec{P}}}_{t}|{{\varvec{P}}}_{0})p_{0t}({{\varvec{A}}}_{t}|{{\varvec{A}}}_{0})$. Thus, according to the separated transition distribution, the new training objectives can be expressed as follows:

13 $$\begin{aligned}&\min _{\theta }\mathbb {E}_{t}\left\{ \gamma _{1}(t)\mathbb {E}_{{{\varvec{M}}}_{0}}\mathbb {E}_{{{\varvec{M}}}_{t}|{{\varvec{M}}}_{0}}\left\| s_{\theta ,t}({{\varvec{M}}}_{t})-\triangledown _{{{\varvec{X}}}_{t}}\text{log}p_{t}({{\varvec{X}}}_{t}|{{\varvec{X}}}_{0}) \right\| _{2}^{2} \right\} \nonumber \\&\min _{\phi }\mathbb {E}_{t}\left\{ \gamma _{2}(t)\mathbb {E}_{{{\varvec{M}}}_{0}}\mathbb {E}_{{{\varvec{M}}}_{t}|{{\varvec{M}}}_{0}}\left\| s_{\phi ,t}({{\varvec{M}}}_{t})-\triangledown _{{{\varvec{P}}}_{t}}\text{log}p_{t}({{\varvec{P}}}_{t}|{{\varvec{P}}}_{0}) \right\| _{2}^{2} \right\} \nonumber \\&\min _{\psi }\mathbb {E}_{t}\left\{ \gamma _{3}(t)\mathbb {E}_{{{\varvec{M}}}_{0}}\mathbb {E}_{{{\varvec{M}}}_{t}|{{\varvec{M}}}_{0}}\left\| s_{\psi ,t}({{\varvec{M}}}_{t})-\triangledown _{{{\varvec{A}}}_{t}}\text{log}p_{t}({{\varvec{A}}}_{t}|{{\varvec{A}}}_{0}) \right\| _{2}^{2} \right\} \end{aligned}$$

In the new optimization objectives, the expectations $\mathbb {E}_{{{\varvec{M}}}_{0}}$ and $\mathbb {E}_{{{\varvec{M}}}_{t}|{{\varvec{M}}}_{0}}$can be efficiently calculated by Monte Carlo estimation of the sample tuple $({{\varvec{M}}}_{0},{{\varvec{M}}}_{t}, t)$. Given that the time-dependent score-based networks described in Eq. 13 can be used to estimate partial scores and the PC sampler can be employed to solve reverse-time SDEs to generate molecules, our task now is to design networks that can efficiently learn the partial scores of 3D geometric structure distribution.

**Fig. 2:**
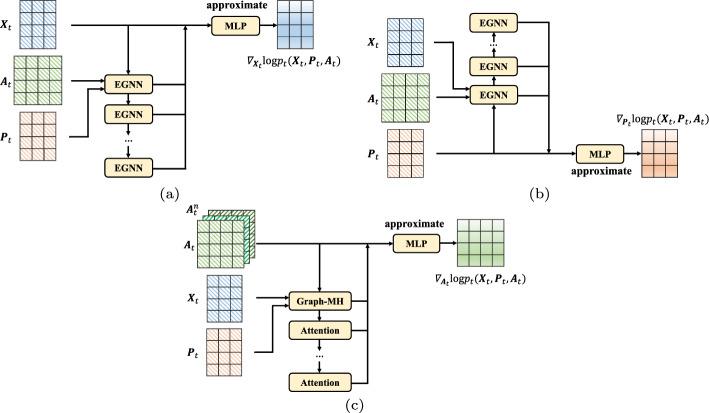
The architecture of the score-based networks used in the proposed method. **a** The score-based network $s_{\theta ,t}$ estimates $\triangledown _{{{\varvec{X}}}_{t}}\text{log}p_{t}({{\varvec{X}}}_{t},{{\varvec{P}}}_{t},{{\varvec{A}}}_{t})$, consisting of EGNN layers and MLP layers. **b** The score-based network $s_{\phi ,t}$ estimates $\triangledown _{{{\varvec{P}}}_{t}}\text{log}p_{t}({{\varvec{X}}}_{t},{{\varvec{P}}}_{t},{{\varvec{A}}}_{t})$, consisting of EGNN layers and MLP layers. **c** The score-based network $s_{\psi ,t}$ estimates $\triangledown _{{{\varvec{A}}}_{t}}\text{log}p_{t}({{\varvec{X}}}_{t},{{\varvec{P}}}_{t},{{\varvec{A}}}_{t})$, consisting of Graph Muti-head Attention layers and MLP layers. The three neural networks take ${{\varvec{X}}}_{t}$, ${{\varvec{P}}}_{t}$, and ${{\varvec{A}}}_{t}$ as input and then estimate the corresponding partial scores, separately

### E(3)-equivariant score-based networks

It has been proved that if the prior distribution is invariant to a group and the networks used to parametrize transition kernels in the diffusion process are equivariant, then the marginal distribution is invariant [12, 13], which is desirable for our 3D molecular generative model. Therefore, we propose a new E(3)-equivariant framework for time-dependent score-based networks, capturing the interdependency among ${{\varvec{X}}}_{t}$, ${{\varvec{P}}}_{t}$, and ${{\varvec{A}}}_{t}$ over time, based on E(n)-equivariant graph neural networks (EGNNs). Furthermore, in order to better model the coordinate variables as well as exploit the spatial information, we incorporate the information of bond angles and torsion angles into score-based networks. The proposed architecture of the score-based networks is illustrated in Fig. 2.

First, we present the score-based network $s_{\theta ,t}$ to estimate $\triangledown _{{{\varvec{X}}}_{t}}\text{log}p_{t}({{\varvec{X}}}_{t},{{\varvec{P}}}_{t},{{\varvec{A}}}_{t})$, and the dimensionality of the network output is consistent with ${{\varvec{X}}}_{t}$. We use multiple layers of EGNNs to learn the partial scores from the atom representations as follows:

14 $$\begin{aligned}&s_{\theta ,t}({{\varvec{G}}}_{t})=\text{MLP}_{\theta }(\left[ {{\varvec{H}}}_{0},{{\varvec{H}}}_{1},...,{{\varvec{H}}}_{L_{H}} \right] ) \nonumber \\&{{\varvec{H}}}_{i+1}=\text{EGNN}({{\varvec{H}}}_{i},{{\varvec{P}}}_{t},{{\varvec{A}}}_{t}) \end{aligned}$$

where ${{\varvec{H}}}_{0} = \left[ {{\varvec{X}}}_{t}, t/T \right]$, $L_{H}$ denotes the number of EGNNs layers, and $\left[ \cdot \right]$ denotes the concatenation operation. By concatenating the node features with *t*/*T* as input to the network, we can better model the time-dependent information.

Then, the score-based network $s_{\phi ,t}$ can be presented to estimate $\triangledown _{{{\varvec{P}}}_{t}}\text{log}p_{t}({{\varvec{X}}}_{t},{{\varvec{P}}}_{t},{{\varvec{A}}}_{t})$ in a similar way, with the output dimensionality consistent with ${{\varvec{P}}}_{t}$. The neural network $s_{\phi ,t}$ is structured as follows:

15 $$\begin{aligned}&s_{\phi ,t}({{\varvec{G}}}_{t})=\text{MLP}_{\phi }(\left[ {{\varvec{R}}}_{0},{{\varvec{R}}}_{1},...,{{\varvec{R}}}_{L_{R}} \right] )\nonumber \\&{{\varvec{R}}}_{i+1}=\text{EGNN}(\left[ {{\varvec{X}}}_{t}, t/T \right] ,{{\varvec{R}}}_{i},{{\varvec{A}}}_{t})-{{\varvec{R}}}_{i} \end{aligned}$$

where ${{\varvec{R}}}_{0} = {{\varvec{P}}}_{t}$, and $L_{R}$ denotes the number of EGNNs layers. The network $s_{\phi ,t}$ needs to be roto-translation equivariant for 3D coordinates, but there is no non-zero distribution that is invariant to translation as it cannot be integrated into one [31]. Thus, we use the distributions on linear subspaces with a constant center of gravity of zero, which have been demonstrated to be consistently used in diffusion [12]. The estimated partial score function $\triangledown _{{{\varvec{P}}}_{t}}\text{log}p_{t}({{\varvec{X}}}_{t},{{\varvec{P}}}_{t},{{\varvec{A}}}_{t})$ is derived from the output of multiple layers of EGNNs, in which the input coordinate ${{\varvec{R}}}_{i}$ is removed at each layer. By subtracting the center of gravity to project the component downward, the output lies on a subspace with a zero center of gravity. Then, the score-based neural networks demonstrate both rotational and reflection equivariance.

Lastly, we will introduce the score-based network $s_{\psi ,t}$. It employs graph multi-head attention to capture crucial relational information between atoms and subsequently utilizes higher-order weighted bond-forming adjacency matrices to learn long-distance dependency as follows:

16 $$\begin{aligned} s_{\psi ,t}({{\varvec{G}}}_{t})=\text{MLP}_{\psi }\left( \left[ \left\{ \text{GraphMH}({{\varvec{H}}}_{i},{{\varvec{P}}}_{i},{{\varvec{A}}}_{t}^{c}) \right\} _{i=0,c=1}^{L_{A},K} \right] \right) \end{aligned}$$

where ${{\varvec{H}}}_{i+1}=\text{EGNN}({{\varvec{H}}}_{i},{{\varvec{P}}}_{t},{{\varvec{A}}}_{t})$ with ${{\varvec{H}}}_{0} = \left[ {{\varvec{X}}}_{t}, t/T \right]$ given, ${{\varvec{R}}}_{i+1}=\text{EGNN}(\left[ {{\varvec{X}}}_{t}, t/T \right] ,{{\varvec{R}}}_{i},{{\varvec{A}}}_{t})-{{\varvec{R}}}_{i}$ with ${{\varvec{R}}}_{0} = {{\varvec{P}}}_{t}$ given, ${{\varvec{A}}}_{t}^{c}$ denotes the *c* sub-higher order matrix of bond-forming adjacency matrix ${{\varvec{A}}}_{t}$ at time *t*, and $L_{A}$ denotes the number of graph multi-head attention layers.

Furthermore, in order to better model the coordinate variables as well as exploit the spatial information, we introduce inter-atomic cosine information into the EGNN layer used in the score-based neural networks. In a 3D molecule with *N* atoms, each node $m_{i}$ is endowed with atom features ${{\varvec{x}}}_i \in \mathbb {R}^{k}$ as well as coordinates ${{\varvec{p}}}_i \in \mathbb {R}^{3}$, and the edge attributes $a_{ij}$ denote the bonding relationship between node $m_{i}$ and $m_{j}$. EGNN consists of *L* equivariant graph convolutional layers (EGCLs), and the new EGCL incorporating angle and torsion angle information in our settings is calculated as follows:

17 $$\begin{aligned}&\mathbf{v}_{ij} = \phi _{angle_{x}}({{\varvec{x}}}_{i}^{l},{{\varvec{x}}}_{j}^{l},d_{ij}^{2},\text{cos}({{\varvec{p}}}_{i}^{l},{{\varvec{p}}}_{j}^{l}),a_{ij} )\nonumber \\&{{\varvec{x}}}_{i}^{l+1}=\phi _{x}({{\varvec{x}}}_{i}^{l},\sum _{j\ne i}^{}\phi _{a}(\mathbf{v}_{ij})\mathbf{v}_{ij} )\nonumber \\&{{\varvec{p}}}_{i}^{l+1}={{\varvec{p}}}_{i}^{l}+\sum _{j\ne i}^{}\frac{{{\varvec{p}}}_{i}^{l}-{{\varvec{p}}}_{j}^{l}}{d_{ij}+1}\phi _{angle_{p}}({{\varvec{x}}}_{i}^{l},{{\varvec{x}}}_{j}^{l},d_{ij}^{2},\text{cos}({{\varvec{p}}}_{i}^{l},{{\varvec{p}}}_{j}^{l}),a_{ij} ) \nonumber \\&{{\varvec{x}}}^{l+1},{{\varvec{p}}}^{l+1}=\text{EGCL}({{\varvec{x}}}^{l},{{\varvec{p}}}^{l},{{\varvec{A}}}) \end{aligned}$$

where $l=0,1,...,L$ indexes the layer, $d_{ij}=\left\| {{\varvec{p}}}_{i}^{l}-{{\varvec{p}}}_{j}^{l} \right\| _{2}$ is the euclidean distance between nodes $m_{i}$ and $m_{j}$, ${{\varvec{A}}} \in \mathbb {R}^{N\times N}$ is the weighted adjacency matrix of 2D graph representation for chemical bonds, and the learnable functions $\phi _{x}$, $\phi _{a}$, $\phi _{angle_{x}}$, and $\phi _{angle_{p}}$ are parameterized by fully connected neural networks. The new EGCL will capture torsion angle information through the learnable functions when aggregating information from all connected neighbors of node $m_{i}$. Notably, since angles and torsion angles are invariant to translation, rotation, and inversion of molecules, this guarantees the equivariance of new EGNN layers as required for 3D molecular generation.

## Experiments results and discussion

This section presents the experimental results of the proposed method EMDS. First, we introduce the benchmark dataset for unconditional 3D molecular generation. Then, we describe the evaluation metrics used in the experiments and analyses. Finally, we compare the performance of our framework with several competitive baseline methods, conduct ablation experiments to highlight the contribution of the individual components in our framework, and present distribution comparisons to demonstrate the effectiveness of our proposed method.

### Experimental setup

We train and evaluate our proposed method on the standard molecule dataset QM9 [16]. QM9^2^ is a widely used molecule dataset that provides information about the various types of atoms, 3D spatial coordinates, atom connectivity (bonding information), and molecular properties for approximately 130,000 small organic molecules. The molecules in the QM9 dataset consist of up to nine heavy atoms (carbon, nitrogen, oxygen, and fluorine) and may have a maximum of 29 atoms, including hydrogen atoms. We use the train/test/validation partitions introduced in previous studies [13, 44], which consist of 100K/13k/18K samples, respectively, for each partition. For a clearer understanding, Table 1 provides specific information about the dataset.

**Table 1:** Statistics of the QM9 dataset

Total Mol Num	Split	Mol Num	Avg Len/Mol	Composition
139885	Train	100k	18.03	H C N O F
	Valid	13K	18.03	
	Test	18K	18.05	

### Evaluation metrics

To evaluate the quality of the generated 3D molecules, we follow [31] and [13], calculating the distance between pairs of atoms and the atom types to predict bond types (single, double, triple, or none). The common metrics considering hydrogen are also adopted in experiments, i.e., the fraction of valid molecules and the fraction of valid and unique molecules. Five metrics considering hydrogen are adopted in experiments:

- **NLL** denotes the negative log likelihood, $\text{log}p({{\varvec{x}}}, {{\varvec{p}}}, {{\varvec{M}}})$. A lower number of NLL indicates better model performance.
- **Validity** indicates the percentage of the generated molecules that follow the chemical valency rules specified by RDkit among all generated molecules.
- **Uniqueness** indicates the percentage of valid & unique molecules among all generated molecules.
- **Atom stability** signifies the proportion of atoms that have the right valency.
- **Molecule stability** signifies the proportion of generated molecules for which all atoms are stable.

### Performance comparison

To evaluate the effectiveness of our method, we compare the proposed model with several competitive models and state-of-the-art models for 3D molecular generation:

- E-NF [31], a molecular generative model equivariant to Euclidean symmetries.
- G-schnet [28], a generative neural network for 3D point sets that respects the rotational invariance of the targeted structures.
- G-spherenet [30], an autoregressive flow model for generating 3D molecular geometries in which invariance and equivariance are ensured.
- GDM-aug [13], a non-equivariant variation generative neural network trained on data augmented with random rotations.
- EDM [13], an E(3) equivariant diffusion model for molecular generation in 3D.
- Molcode [45], a roto-translation equivariant generative framework for molecular graph-structure co-design.

We conduct comparative experiments using publicly available code for these baselines, generate 1000 samples for each model, and present the experimental results thereafter.

**Fig. 3:**
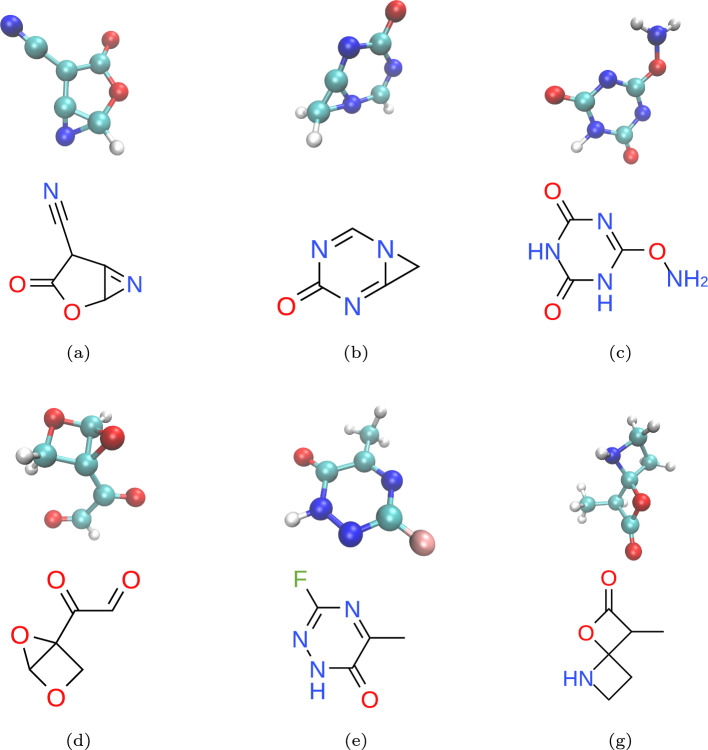
Visualization of molecules generated by the proposed method. The 2D molecular graphs are shown below their corresponding 3D geometries

The comparative results of de novo molecular generation on the QM9 dataset are presented in Table 2. Some visualizations (2D and 3D) of the molecules generated by our proposed model are shown in Fig. 3. The proposed method is run three times, and the mean performance is reported.

**Table 2:** Performance on the QM9 dataset with explicit hydrogen atoms

Method	NLL	Valid (%)	Valid & unique(%)	Atom Sta (%)	Mol Sta (%)
E-NF	$-$59.7	40.2	39.4	85.0	4.9
G-Schnet	N.A	85.5	80.3	95.7	68.1
G-Spherenet	N.A	88.1	82.8	67.2	13.4
GDM-aug	$-$92.5	90.4	89.5	97.6	71.6
EDM	$-$110.7	91.9	90.7	98.7	82.0
Molcode	N.A	94.1	91.1	98.6	83.6
EMDS(w/o eq)	$-$97.4	87.3	83.5	97.3	72.0
EMDS(w/o cos)	$-$127.7	93.5	90.3	98.4	82.3
EMDS(w fsn)	$-$130.8	93.9	91.2	98.5	83.3
EMDS	$-$**137.1**	**95.8**	**93.6**	**99.2**	**89.2**

Bold values indicate evaluation metrics and the best results among all the methods

In Table 2, we see that our proposed model EMDS outperforms other generative methods, i.e., E-NF, G-Schnet, G-Spherenet, Molcode, and EDM, as well as its non-equivariant counterpart GDM-aug for all metrics. The proposed model generates the highest percentage of valid and valid & unique molecules compared with all other methods, outperforming previous results in such measures by at least 1.7% and 2.5%, respectively. The proposed model is able to generate a very high rate of atom stability and molecule stability, outperforming other results in such measures by at least 0.5% and 5.6%, respectively. In particular, it also advances the other results in terms of negative log-likelihood (NLL) by at least 23%, which suggests that even under a similar diffusion modeling framework, the generative distribution learned by the proposed method may contain much sharper peaks. Moreover, the excellent NLL results of EMDS also demonstrate its better effectiveness in decomposing the complete diffusion process into diffusion processes of individual components of the molecular feature space compared with uniform Gaussian diffusion methods. The comparative results demonstrate the effectiveness and excellent prospects of the proposed method for 3D molecular generation.

### Ablation experiments

To further analyze the impact of certain key model components within the proposed method, we also include three variants of the proposed method for ablation studies. The three variants include the proposed method that uses a non-equivariant graph network [46] (i.e., EMDS w/o eq), the proposed method without the inter-atomic cosine information of angles and torsion angles applied to networks (i.e., EMDS w/o cos), and the proposed method using a single network instead of obtaining the partial score functions of ${{\varvec{X}}}_{t}$ and ${{\varvec{P}}}_{t}$ separately, which uses fewer score-based networks (i.e., EMDS w fsn). The results of the ablation studies are presented in Table 2.

When using a single network to obtain the partial score functions of ${{\varvec{X}}}_{t}$ and ${{\varvec{P}}}_{t}$, the effectiveness of the variant with fewer score-based networks in generating molecules is significantly lower. In particular, EMDS outperforms the variant by 5.9% in terms of molecule stability. The results suggest that the complex dependencies among the components of the molecular feature space significantly affect the stability of the generated molecules. The proposed model structure builds three score-based networks for each component to be modeled, effectively capturing and learning the complex dependencies and greatly facilitating molecular generation.

Compared with the variant using the non-equivariant network, the performance improvement of our method is significant in all metrics, proving the effectiveness of our proposed equivariant geometric structure diffusion framework. And compared the variant w/o cos, the proposed method performs much better in all metrics, especially molecular stability with a significant improvement of 6.9%. We speculate that the reason for the performance gain is that the inter-atomic cosine information contains information of angles and torsion angles, which implicitly determines the spatial coordinates of the molecules, and our proposed method can effectively capture and learn the information to contribute to a clear improvement.

**Fig. 4:**
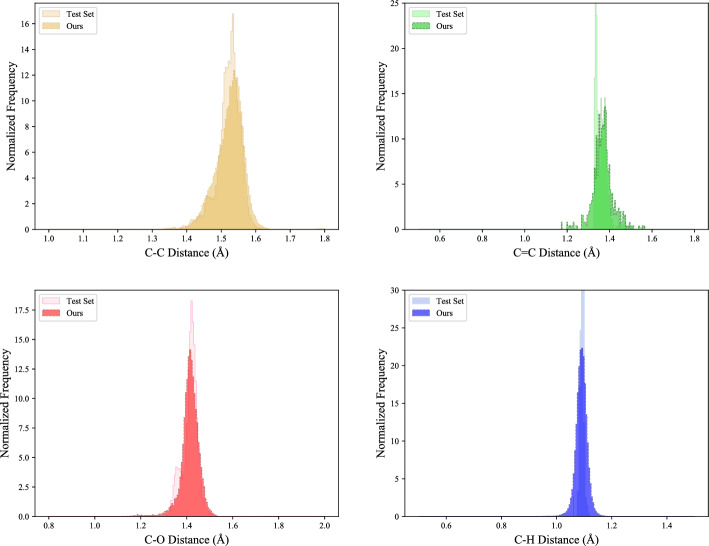
Distribution comparison of bond lengths between molecules in the test set and molecules generated by the proposed method

**Fig. 5:**
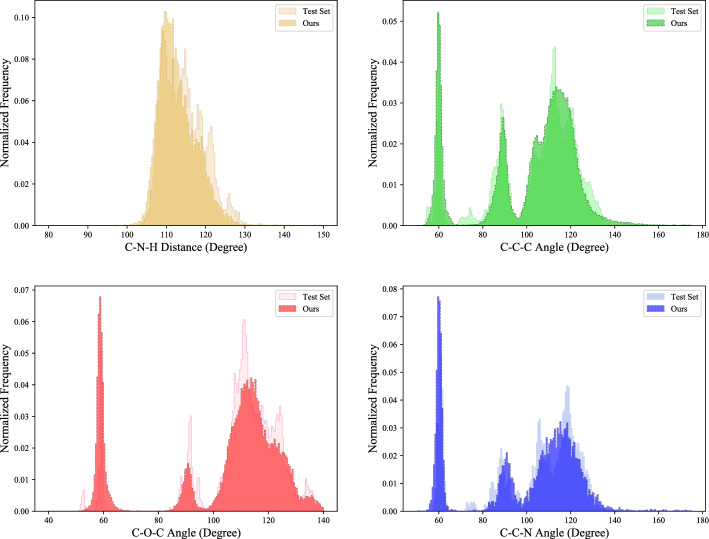
Distribution comparison of bond angles between molecules in the test set and molecules generated by the proposed method

**Fig. 6:**
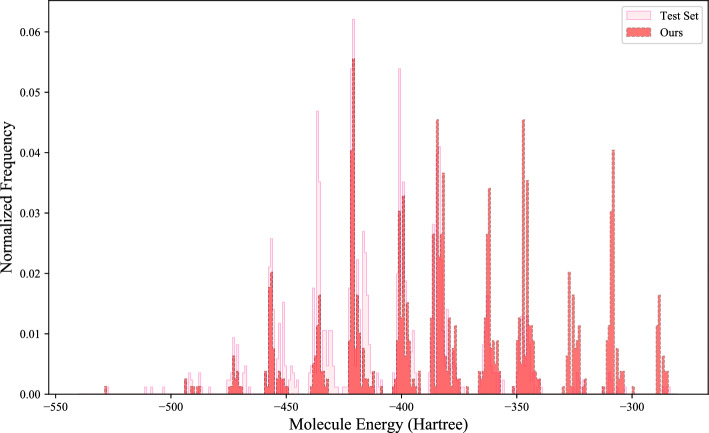
Distribution comparison of potential energy between molecules in the test set and molecules generated by the proposed method

### Distribution comparisons

We further test the performance of our model in generating 3D geometric molecules by comparing the distributions of common bond lengths, bond angles, and potential energy in the test set and the generated samples (Figs. 4, 5, and 6). In Fig. 4, differences in distance distribution between various types of bond lengths are evident, which are effectively captured in our model. The performance difference mainly emanates from certain distributions with higher peaks where bond lengths are more stable in a smaller value range. As seen in Fig. 5, our model excellently represents the distributions of bond angles, including those with multiple peaks. And in Fig. 6, it can be observed that the potential energy distribution of the generated molecules is in good agreement with that of the QM9 dataset, demonstrating the molecules generated by the proposed method have good geometrical structural properties. These results show that our method can efficiently generate molecules that are close to the real situation and has excellent capacity for modeling chemically realistic molecules.

## Conclusion

In this paper, we propose EMDS, a novel E(3)-equivariant score-based diffusion framework for 3D molecular generation. Our approach implements a diffusion process via SDEs to incorporate 3D geometric structure, in which the full diffusion is decomposed into diffusion processes of respective components of the molecular feature space, thus overcoming the limitations of the traditional Gaussian diffusion methods. Furthermore, we also consider the angle and torsion angle information, which is naturally translation-invariant, for improved modeling of molecular coordinates as well as exploiting the spatial information. Experiments and comparative results have demonstrated that our framework clearly outperforms previous 3D molecular generation methods and has significantly better capacity for modeling chemically realistic molecules.

Beyond the work presented here, the scenarios closer to real biomedicine (e.g., pocket-conditioned generation, RNA structure matching, and targeted molecule generation) are clearly of future research interest in molecule design. We plan to explore the prospect of expanding EMDS to the various 3D geometry generation applications in challenging biomedical scenarios.

## Data Availability

We make the dataset and source code publicly available at https://github.com/nclabhzhang/EMDS. And the dataset used in this work is directly available for download at https://deepchemdata.s3-us-west-1.amazonaws.com/datasets/gdb9.tar.gz.

## Footnotes

1 We follow the convention to use $\text{R} x$ to denote applying group actions $\text{R}$ on $x$, which formally is calculated as $x \text{R}^{ T }$.

2 http://dx.doi.org/10.6084/m9.figshare.978904.

## References

[1] Zhao B-W, Su X-R, Hu P-W, Ma Y-P, Zhou X, Hu L. A geometric deep learning framework for drug repositioning over heterogeneous information networks. Brief Bioinform. 2022;23(6):384. 10.1093/bib/bbac384. 10.1093/bib/bbac384 36125202

[2] Zhao B-W, Su X-R, Hu P-W, Huang Y-A, You Z-H, Hu L. iGRLDTI: an improved graph representation learning method for predicting drug-target interactions over heterogeneous biological information network. Bioinformatics. 2023;39(8):451. 10.1093/bioinformatics/btad451. 10.1093/bioinformatics/btad451 PMC10397422 37505483

[3] Simonovsky M, Komodakis N. Graphvae: towards generation of small graphs using variational autoencoders. In: Artificial neural networks and machine learning–ICANN 2018: 27th international conference on artificial neural networks, Rhodes, Greece, October 4–7, 2018, Proceedings, Part I 27, 412–422 (2018). Springer.

[4] Luo S, Guan J, Ma J, Peng J. A 3d generative model for structure-based drug design. Adv Neural Inf Process Syst. 2021;34:6229–39.

[5] Simm GN, Pinsler R, Csányi G, Hernández-Lobato JM. Symmetry-aware actor-critic for 3d molecular design. arXiv preprint arXiv:2011.12747, 2020.

[6] Ho J, Jain A, Abbeel P. Denoising diffusion probabilistic models. Adv Neural Inf Process Syst. 2020;33:6840–51.

[7] Song Y, Sohl-Dickstein J, Kingma DP, Kumar A, Ermon S, Poole B. Score-based generative modeling through stochastic differential equations. In: International conference on learning representations, 2021.

[8] Jo J, Lee S, Hwang SJ. Score-based generative modeling of graphs via the system of stochastic differential equations. In: International conference on machine learning, 2022;10362–10383. PMLR.

[9] Vignac C, Krawczuk I, Siraudin A, Wang B, Cevher V, Frossard P. Digress: discrete denoising diffusion for graph generation. In: Proceedings of the 11th international conference on learning representations, 2023.

[10] Shi C, Luo S, Xu M, Tang J. Learning gradient fields for molecular conformation generation. In: International conference on machine learning, 2021;9558–9568. PMLR.

[11] Luo S, Shi C, Xu M, Tang J. Predicting molecular conformation via dynamic graph score matching. Adv Neural Inf Process Syst. 2021;34:19784–95.

[12] Xu M, Yu L, Song Y, Shi C, Ermon S, Tang J. Geodiff: a geometric diffusion model for molecular conformation generation. arXiv preprint arXiv:2203.02923, 2022.

[13] Hoogeboom E, Satorras VG, Vignac C, Welling M. Equivariant diffusion for molecule generation in 3d. In: International conference on machine learning, 2022;8867–8887. PMLR.

[14] Wu L, Gong C, Liu X, Ye M, Liu Q. Diffusion-based molecule generation with informative prior bridges. Adv Neural Inf Process Syst. 2022;35:36533–45.

[15] Vignac C, Osman N, Toni L, Frossard P. Midi: mixed graph and 3d denoising diffusion for molecule generation. arXiv preprint arXiv:2302.09048, 2023.

[16] Ramakrishnan R, Dral PO, Rupp M, Von Lilienfeld OA. Quantum chemistry structures and properties of 134 kilo molecules. Scientific Data. 2014;1(1):1–7. 10.1038/sdata.2014.22 PMC4322582 25977779

[17] Zhang M, Jiang S, Cui Z, Garnett R, Chen Y. D-vae: A variational autoencoder for directed acyclic graphs. Advances in Neural Information Processing Systems. 2019;32.

[18] Popova M, Shvets M, Oliva J, Isayev O. Molecularrnn: generating realistic molecular graphs with optimized properties. arXiv preprint arXiv:1905.13372, 2019.

[19] Zhou Z, Kearnes S, Li L, Zare RN, Riley P. Optimization of molecules via deep reinforcement learning. Sci Rep. 2019;9(1):10752. 31341196 10.1038/s41598-019-47148-x PMC6656766

[20] Lim J, Hwang S-Y, Moon S, Kim S, Kim WY. Scaffold-based molecular design with a graph generative model. Chem Sci. 2020;11(4):1153–64. 10.1039/c9sc04503a PMC8146476 34084372

[21] Weininger D. Smiles, a chemical language and information system. 1. introduction to methodology and encoding rules. J Chem Inf Comput Sci. 1988;28(1):31–6.

[22] Kusner MJ, Paige B, Hernández-Lobato JM. Grammar variational autoencoder. In: International conference on machine learning, 2017;1945–1954. PMLR.

[23] Segler MH, Kogej T, Tyrchan C, Waller MP. Generating focused molecule libraries for drug discovery with recurrent neural networks. ACS Cent Sci. 2018;4(1):120–31. 29392184 10.1021/acscentsci.7b00512 PMC5785775

[24] Gómez-Bombarelli R, Wei JN, Duvenaud D, Hernández-Lobato JM, Sánchez-Lengeling B, Sheberla D, Aguilera-Iparraguirre J, Hirzel TD, Adams RP, Aspuru-Guzik A. Automatic chemical design using a data-driven continuous representation of molecules. ACS Cent Sci. 2018;4(2):268–76. 29532027 10.1021/acscentsci.7b00572 PMC5833007

[25] You J, Liu B, Ying Z, Pande V, Leskovec J. Graph convolutional policy network for goal-directed molecular graph generation. Advances in neural information processing systems. 2018;31.

[26] Jin W, Barzilay R, Jaakkola T. Junction tree variational autoencoder for molecular graph generation. In: International Conference on Machine Learning, 2018;2323–2332. PMLR.

[27] Shi C, Xu M, Zhu Z, Zhang W, Zhang M, Tang J. Graphaf: a flow-based autoregressive model for molecular graph generation. In: International Conference on Learning Representations, 2019.

[28] Gebauer N, Gastegger M, Schütt K. Symmetry-adapted generation of 3d point sets for the targeted discovery of molecules. Advances in neural information processing systems. 2019;32.

[29] Gebauer NW, Gastegger M, Hessmann SS, Müller K-R, Schütt KT. Inverse design of 3d molecular structures with conditional generative neural networks. Nat Commun. 2022;13(1):973. 35190542 10.1038/s41467-022-28526-y PMC8861047

[30] Luo Y, Ji S. An autoregressive flow model for 3d molecular geometry generation from scratch. In: International conference on learning representations (ICLR) 2022.

[31] Garcia Satorras V, Hoogeboom E, Fuchs F, Posner I, Welling M. E (n) equivariant normalizing flows. Adv Neural Inf Process Syst. 2021;34:4181–92.

[32] Kingma D, Salimans T, Poole B, Ho J. Variational diffusion models. Adv Neural Inf Process Syst. 2021;34:21696–707.

[33] Anand N, Achim T. Protein structure and sequence generation with equivariant denoising diffusion probabilistic models. arXiv preprint arXiv:2205.15019, 2022.

[34] Peng X, Guan J, Liu Q, Ma J. Moldiff: addressing the atom-bond inconsistency problem in 3d molecule diffusion generation. arXiv preprint arXiv:2305.07508 2023.

[35] Lin H, Huang Y, Zhang O, Liu Y, Wu L, Li S, Chen Z, Li SZ. Functional-group-based diffusion for pocket-specific molecule generation and elaboration. Advances in Neural Information Processing Systems. 2024;36.

[36] Igashov I, Stärk H, Vignac C, Satorras VG, Frossard P, Welling M, Bronstein M, Correia B. Equivariant 3d-conditional diffusion models for molecular linker design. arXiv preprint arXiv:2210.05274 2022.

[37] Sohl-Dickstein J, Weiss E, Maheswaranathan N, Ganguli S. Deep unsupervised learning using nonequilibrium thermodynamics. In: International conference on machine learning, 2015;2256–2265. PMLR.

[38] Nichol AQ, Dhariwal P. Improved denoising diffusion probabilistic models. In: International conference on machine learning, 2021;8162–8171. PMLR.

[39] Kingma DP, Salimans T, Jozefowicz R, Chen X, Sutskever I, Welling M. Improved variational inference with inverse autoregressive flow. Advances in neural information processing systems 2016;29.

[40] Sønderby CK, Raiko T, Maaløe L, Sønderby SK, Winther O. Ladder variational autoencoders. Advances in neural information processing systems 2016:29.

[41] Köhler J, Klein L, Noé F. Equivariant flows: exact likelihood generative learning for symmetric densities. In: International conference on machine learning, 2020;5361–5370. PMLR.

[42] Kloeden PE, Platen E. Stochastic differential equations. Berlin and Heidelberg: Springer; 1992. p. 103–60.

[43] Grenander U, Miller MI. Representations of knowledge in complex systems. J Roy Stat Soc: Ser B (Methodol). 1994;56(4):549–81.

[44] Anderson B, Hy TS, Kondor R. Cormorant: covariant molecular neural networks. Advances in neural information processing systems 2019;32.

[45] Zhang Z, Liu Q, Lee C-K, Hsieh C-Y, Chen E. An equivariant generative framework for molecular graph-structure co-design. Chem Sci. 2023;14(31):8380–92. 37564414 10.1039/d3sc02538a PMC10411624

[46] Gilmer J, Schoenholz SS, Riley PF, Vinyals O, Dahl GE. Neural message passing for quantum chemistry. In: International conference on machine learning, 2017;1263–1272. PMLR.

